# Anti-dopaminergic effect of the methanolic extract of *Morus alba* L. leaves

**DOI:** 10.4103/0253-7613.44154

**Published:** 2008-10

**Authors:** Adhikrao V. Yadav, Vandana S. Nade

**Affiliations:** Government College of Pharmacy, Vidyanagar, Karad, Satara, India; 1 Department of Pharmacology, N. D. M. V. P. Samaj's College of Pharmacy, Nashik, India

**Keywords:** Anti-dopaminergic, catalepsy, *Morus alba* L, stereotypy, vas deferens

## Abstract

**Objective:**

To evaluate the effect of methanolic extract of *Morus alba* L. leaves on dopaminergic function.

**Materials and Methods:**

The effect of the methanolic extract of *Morus alba* L. leaves was evaluated on haloperidol and metoclopramide induced catalepsy, foot shock-induced aggression, amphetamine-induced stereotyped behavior and phenobarbitone induced sleeping in mice. In each of these tests, the extract was administered in doses of 50, 100 and 200 mg/kg, i.p., 30 min before performing the test in mice. Further, the inhibitory effect of the extract on dopamine was studied using isolated rat vas deferens.

**Results:**

The extract produced significant dose dependent potentiation of haloperidol (1 mg/kg, i.p.) and metoclopramide (20 mg/kg, i.p.) induced catalepsy in mice. The extract significantly reduced number of fights and increased latency to fights in foot shock-induced aggression; it also decreased amphetamine (1 mg/kg, i.p.) induced stereotyped behavior in a dose dependent manner. The sleeping time induced by phenobarbitone (50 mg/kg, i.p.) too was prolonged. The extract inhibited contractions produced by dopamine on isolated rat vas deferens.

**Conclusion:**

The results suggest that the methanolic extract of *Morus alba* L. possesses antidopaminergic activity. Further neurochemical investigation can explore the mechanism of action of the plant drug with respect to antidopaminergic functions and help to establish the plant as an antipsychotic agent.

## Introduction

*Morus alba* L. (Moraceae) (MA) is a moderately sized tree, three to six metres high, native of India, China and Japan. It is occasionally cultivated elsewhere in Europe, North America, and Africa. *Morus alba* is commonly known as white mulberry. White mulberry is cultivated throughout the world, wherever silkworms are raised. The leaves of white mulberry is the main food source for the silkworms.[1]

White mulberry has a long history of medicinal use in Chinese medicine. Almost all the parts of the plant are used as medicine.[2] Traditionally, the mulberry fruit has been used as a medicinal agent to nourish the blood, benefit the kidneys and treat weakness, fatigue, anemia and premature graying of hair. It is also used to treat urinary incontinence, tinnitus, dizziness and constipation in the elderly patient.[3] The medicinal uses of the plant reported so far include analgesic, antiasthmatic, antirheumatic, antitussive, astringent, diaphoretic, diuretic, emollient and expectorant, hypotensive and brain tonic.[1–4] The plant extract has been demonstrated to posses free radical scavenging activity.[5] Hypoglycemic and antioxidant potency of some phenolic compounds (Flavonoids, stilbenes and 2-arylbenzofurans) have been reported from MA.[6] Besides, MA has been known to show antiviral and antimicrobial effect.[7] The plant has been extensively studied for its hypolipidemic,[8] neuroprotective,[9] hepatoprotective,[10] hypouricemic,[11] and cardioprotective actions.[12] The plant is reported to contain the phytoconstituent tannins, phytosterols, sitosterols, saponins, triterpenes, flavanoids, benzofuran derivatives, morusimic acid, anthocyanins, anthroquinones, glycosides and oleanolic acid as the main active principles.[13–15]

Although several medicinal uses have been reported for MA, no investigative report pertaining to its central nervous system activity exists. Hence, an attempt has been made to evaluate the antidopaminergic activity of the plant.

## Materials and Methods

### Extract preparation

Fresh leaves of the plant were collected in the month of October, from a local area in Nashik, India, and authenticated by P. S. N. Rao (Director, Botanical Survey of India, Pune). A voucher specimen of the plant has been deposited at the Botanical Survey of India, Pune (Voucher Specimen No. NVMA2). The leaves were washed and cut into pieces and air dried. The powdered plant material was defatted using petroleum ether (60 - 80°C), using a Soxhlet extractor (Space Lab, Nashik, India). The marc was further extracted using methanol for 72 h, to obtain the extract. The extract was filtered and evaporated to dryness on a rotary evaporator, under reduced pressure (Space Lab, Nashik, India). The yield of methanolic extract of *Morus alba* L. (MAE) leaves was found to be 2.2% w/w. Before use, the extract was dissolved in distilled water, for administration intraperitoneally (i.p.).

### Phytochemical screening

Phytochemical investigations of the extract were carried out using the methods described by Kokate, Trease and Evans,[16,17] to check for the presence of phenolic compounds, flavonoids, tannins, triterpenes, anthocyanins, anthroquinones and sterols. The presence of alkaloids and saponins was also ascertained.

### Animals

Albino male Swiss mice (18 - 22 g) and male Wistar rats (180 - 220 g) were used for the study. The animals were housed in colony cages and maintained under the standard environmental conditions - temperature 25 ± 2°C, 12 h light : 12 h dark cycle and 50 ± 5 % relative humidity, with food and water *adlibitum*. The animals were deprived of food the night before the experiment and during the experiment. All experiments were carried out during the light period (08.00 -16.00 h). The Institutional Animal Ethical Committee of N.D.M.V.P.S College of Pharmacy, Nashik approved the protocol of the study (CPN/IAEC/2007/01).

### Drugs

All the drug solutions *viz.* Haloperidol (RPG Life Sciences, India), metoclopramide (Ipca Lab, India), d-amphetamine (Sigma, USA), phenobarbitone (Samarth Life Sciences, India) and Dopamine HCl (VHP Life Sciences, India), were prepared in distilled water.

### Acute toxicity test

The extract was administered orally and i.p. in doses of 50, 100, 200, 500, 1000, 1500 and 2000 mg/kg to different groups of mice, each group consisting of six animals (n = 6). The mortality rate was observed and recorded for a 24-hour period.

### Haloperidol-induced catalepsy

Haloperidol (1 mg/kg) was injected intraperitoneally to mice (n = 6) pretreated with vehicle (10 ml/kg, i.p.) or MAE (50, 100 and 200 mg/kg, i.p.). The vehicle or MAE was administered 30 min prior to the administration of haloperidol. The duration of catalepsy was measured at 0, 30, 60, 90, 120, 150 and 180 min, using Bar test.[18] Both the forepaws of mouse were placed on a horizontal bar raised 3 cm from the table, and the time required to remove the forepaws from the bar was recorded as the duration of catalepsy. In all the experiments, the observer was blind to the treatment given to the mice. Between experiments, the animals were returned to their home cages.

### Metoclopramide - induced catalepsy

Metoclopramide (20 mg/kg) was injected intraperitoneally to mice (n = 6) pretreated with vehicle (10 ml/kg, i.p.) or MAE (50, 100 and 200 mg/kg, i.p.) and the duration of catalepsy was measured as described in haloperidol-induced catalepsy.[19–21]

### Foot shock-induced aggression

Foot shock-induced aggression (FSIA) behavior was induced in pairs of mice by administering a train of impulses through an electronic stimulator to a grid floor, for three minutes. The animals were divided into five groups of 12 mice (six pairs of male mice) per group. The vehicle (10 ml/kg, i.p.), haloperidol (1 mg/kg, i.p.) as a standard and MAE (50, 100 and 200 mg/kg) were administered i.p., 30 min prior to the experiment. Aggressive behavior was noted in pairs of mice by using two parameters *viz.* number of fights and latency to fight.[22]

### Amphetamine-induced stereotyped behavior in mice

The mice were allowed a maximum of 30 min to get acclimatized to the observation cage, prior to the experiment. Amphetamine-induced stereotypy was scored blind by an independent observer, every five minutes for 30 min. Stereotypy scoring - 0, absence of stereotyped behavior; 1, intermittent sniffing; 2, constant sniffing; 3, constant sniffing with intermittent licking and/or false biting; 4, constant licking or false licking; 5, constant licking; 6, constant biting and moving around; 7, constant biting and restricted to a small area in the cage; 8, rearing - was used.

The animals were divided into five groups, each containing six animals. They were treated with vehicle (10 ml/kg, i.p.) or extract 50, 100 and 200 mg/kg and placed individually in the cage. Amphetamine (1 mg/kg i.p.) was given 30 minutes after the extract was administered. The stereotyped behavior was recorded.[23]

### Phenobarbitone-induced sleeping time in mice

Phenobarbitone (50 mg/kg) was injected i.p. to mice (n = 6) pretreated with vehicle (10 ml/kg, i.p.) or MAE 50, 100 and 200 mg/kg. Haloperidol (1 mg/kg, i.p.) was used as positive control. The vehicle, MAE and haloperidol were administered 30 minutes prior to the administration of phenobarbitone. Immediately after the phenobarbitone administration, each animal was placed in an individual cage and observed. The latency to the loss of righting reflex (induction time in min) and the time required to recover righting reflex or awakening (sleeping time in min) were noted for each animal.[24,25]

### Effect of MAE on dopamine-induced contraction of isolated rat vas deferens

Adult male Wistar rats were sacrificed by cervical dislocation and the vas deferens was removed and kept in Krebs-Henseleit solution. The concentration related contraction response (CRC) to dopamine (10, 20, 40, 80 and 160 *µ*g/ml) was recorded on Sherrigton rotating drum (INCO, Ambala, India). Concentration related contraction response to dopamine was later repeated in the presence of MAE (0.5 ml of 25 mg/ml). The contact time between the dopamine and the tissue was maintained at 60 seconds.[26]

### Statistical analysis

Results are expressed as mean ± S.E.M. and the statistical analysis of data was done using one-way analysis of variance (ANOVA), followed by Dunnett's test. Probability level less than 0.05 was considered statistically significant.

## Results

### Phytochemical screening

The phytochemical screening of MAE revealed the presence of phenolic compounds, flavonoids, tannins, sterols, alkaloids and saponins.

### Acute toxicity test

Oral and i.p. administration of MAE up to 2 g/kg did not produce any toxic effects in the mice. No mortality was observed and MAE was found to be safe at given doses.

### Haloperidol-induced catalepsy

**Figure 1:**
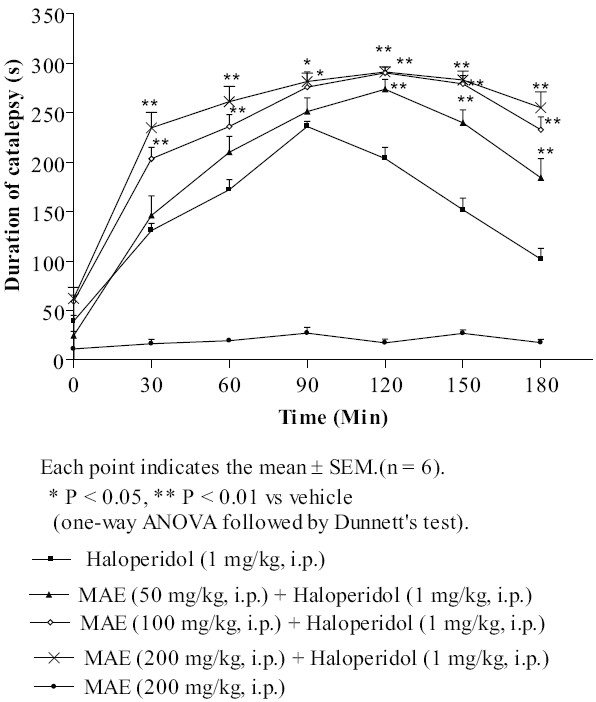
Effect of MAE on haloperidol-induced catalepsy

In vehicle treated animals, haloperidol (1 mg/kg. i.p.) produced the maximum catalepsy at 90 min (236.2 ± 5.275 s). Methanolic extract of *Morus alba* L. (50, 100, 200 mg/kg, i.p.) significantly potentiated haloperidol induced catalepsy at each time interval, in a dose dependent manner. At dose 50, 100 and 200 mg/kg, MAE showed maximum cataleptic score 275.8 ± 9.998, 290.3 ± 5.852 and 291.2 ± 5.288 s, respectively at 120 min (P < 0.01) in haloperidol treated animals. The mice treated with MAE (200 mg/kg, i.p.) did not exhibit any catalepsy and appeared the same as the normal animals [Figure 1].

### Metoclopramide-induced catalepsy

**Figure 2:**
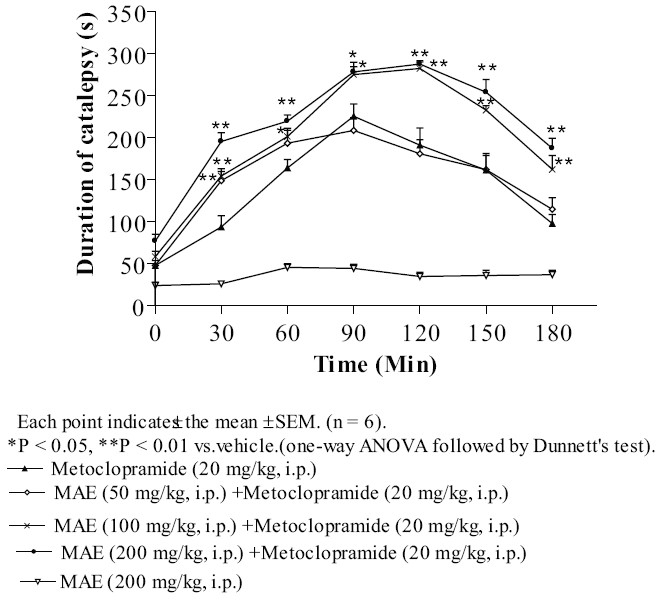
Effect of MAE on metoclopramide-induced catalepsy in mice

The animals treated with MAE 200 mg/kg i.p. did not exhibit any obvious behavioral syndrome and catalepsy. Pretreatment with MAE (100 and 200 mg/kg, i.p.) significantly potentiated the cataleptic effect of metoclopramide (20 mg/kg, i.p.) at each time interval; however, MAE 50 mg/kg, i.p did not show statistically significant potentiation [Figure 2].

### Foot shock-induced aggression

**Figure 3:**
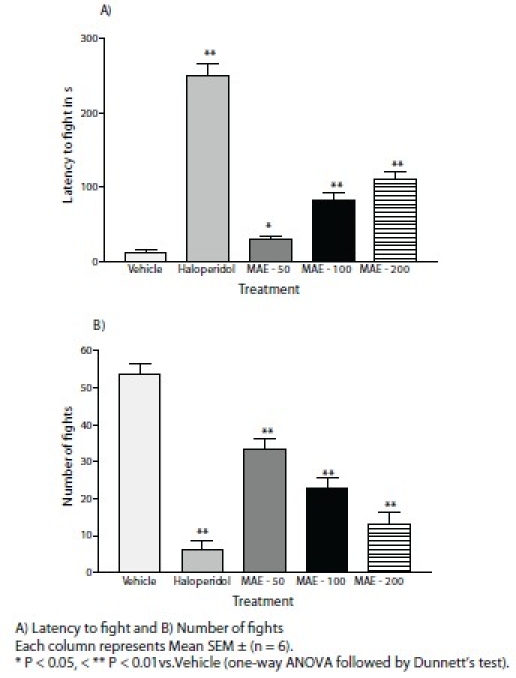
Effect of MAE on foot shock-induced aggression

The intraperitoneal administration of MAE (50, 100 and 200 mg/kg) showed a significant (*P* < 0.01) dose dependent decrease in the number of fights in foot shock-induced aggression, as compared with vehicle. Further MAE (100 and 200 mg/kg, i.p.) significantly (*P* < 0.01) increased latency to fight, but 50 mg/kg did not produce any significant increase. The haloperidol (1 mg/kg, i.p.) treated group showed a statistically significant (*P* < 0.01) decrease in the number of fights and an increase in the latency to fight. [Figure  3A and B].

### Amphetamine-induced stereotyped behavior in mice

**Figure 4:**
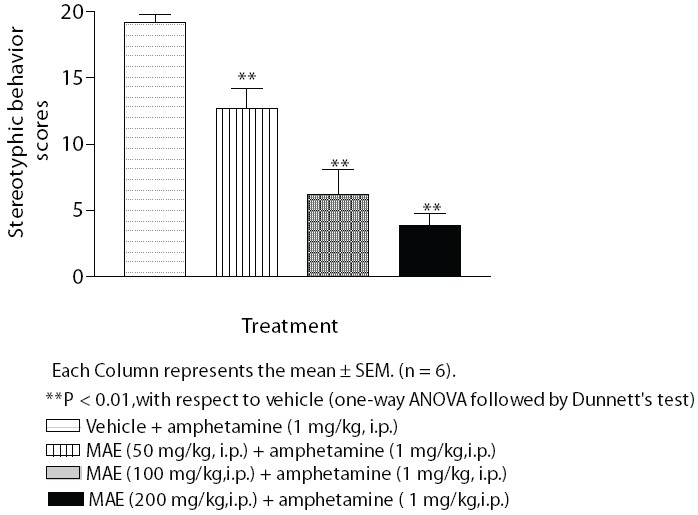
Effect of MAE on amphetamine (1 mg/kg) induced stereotyped behaviour in mice

Amphetamine (1 mg/kg) induced a stereotyped behavior characterized by intermittent sniffing or constant sniffing, licking, biting, moving around, restricted to a small area in the cage and rearing. Administration of MAE (50, 100 and 200 mg/kg, i.p.) significantly (*P* < 0.01) decreased amphetamine-induced stereotyped behavior [Figure 4].

### Phenobarbitone-induced sleeping time in mice

**Figure 5:**
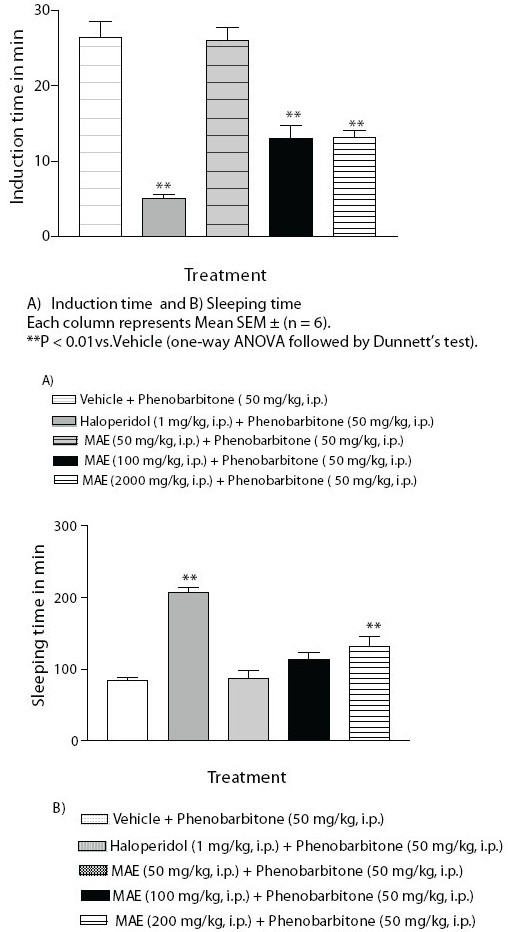
Effect of MAE on phenobarbitone induced sleeping time

Pretreatment with haloperidol (1 mg/kg, i.p.) and MAE (200 mg/kg, i.p.) produced significant prolongation of phenobarbitone (50 mg/kg, i.p.) induced sleeping time. Phenobarbitone-induced sleeping time was also prolonged by MAE (100 mg/kg, i.p.), though it was not statistically significant. Further haloperidol and MAE (100 and 200 mg/kg) significantly reduced the induction time of sleep. *Morus alba* L. in dose 50 mg/kg did not alter induction and sleeping time, as compared to the vehicle group [Figure 5A and B].

### Effect of MAE on dopamine-induced contraction of isolated rat vas deferens

**Figure 6:**
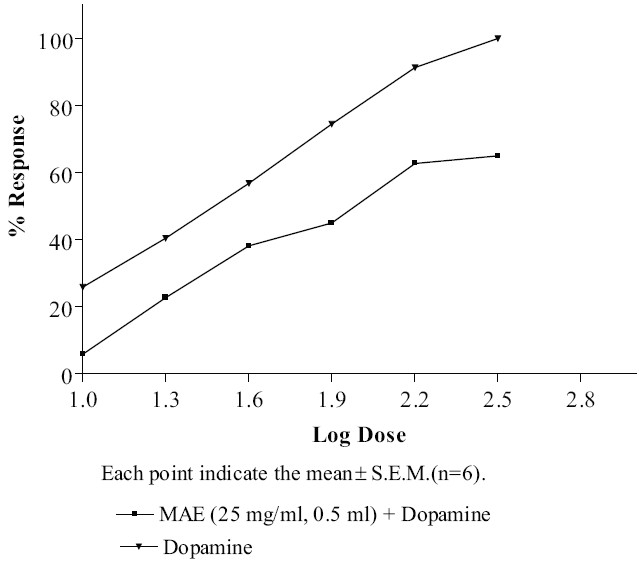
Effect of MAE on dopamine - induced contraction of isolated rat vas deferens

Dopamine produced concentration related contractile response in rat vas deferens. *Morus alba* L. reduced the contraction produced by dopamine on rat vas deferens [Figure 6].

## Discussion

Haloperidol, a typical neuroleptic produces catalepsy in rodents and extrapyramidal side effects in human.[27] Haloperidol-induced catalepsy is one of the animal models for testing the extrapyramidal side effects of antipsychotic drugs. Haloperidol, (a non-selective D_2_ dopamine antagonist) and metoclopramide (a potent dopaminergic blocking agent) induced catalepsy is primarily due to blockade of dopamine receptors in the striatum. The agents increasing dopamine transmission inhibits neuroleptic-induced catalepsy. The striatum and nucleus accumbens have been implicated as the major brain structures involved in antipsychotic induced catalepsy, which appears due to the blockade of dopamine neurotransmission.[28] In the present study, MAE (50, 100 and 200 mg/kg, i.p.) significantly (*P* < 0.05, *P* < 0.01) potentiated dose dependent haloperidol- and metoclopramide-induced catalepsy. Thus, the results suggest that MAE shows antidopaminergic activity.

Central monoaminergic neurons appear to play an essential role in the modulation of aggressive behavior. Central D_2_ dopamine receptors are involved in the modulation of foot shock-induced aggression in mice. Brain dopamine level is increased on foot shock-induced aggression.[22] Methanolic extract of *Morus alba* L. (50, 100 and 200 mg/kg, i.p.) significantly (*P* < 0.01) reduced the number of fighting attacks and increased the latency to fight, indicating antidopaminergic activity in FSIA.

Amphetamine-induced stereotyped behavior is a well established model for schizophrenia.[29] Amphetamine is an indirectly acting sympathomimetic agent, which releases dopamine and induces characteristic stereotyped behavior.[22] Amphetamine-induced stereotyped behavior is a measure of dopamine D_2_ receptor reactivity. It is known that amphetamine-induced stereotyped behavior is mediated by the hyperactivity of dopaminergic mechanism in the nigrostriatal and mesolimbic pathway.[26] Methanolic extract of *Morus alba* L. (50, 100 and 200 mg/kg, i.p.) significantly (*P* < 0.01) blocked amphetamine-induced stereotyped behavior in mice. This suggests that the extract contains antidopaminergic compound(s). A majority of antipsychotic drugs (phenothiazines, butyrophenones) used in the management of psychosis are known to have preference for D_2_ receptor and abolish amphetamine-induced stereotyped behavior. Since MAE has antidopaminergic potential, it needs further investigations in the treatment of psychosis.

Earlier reports of chemical constituents and their pharmacology suggest that the plants containing flavonoids, saponins and tannins possess activity against many CNS disorders.[30,31] Phytochemical tests of MAE revealed the presence of flavonoids, saponins and tannins, which may be responsible for the antidopaminergic potential of the extract.

Drug decreasing dopaminergic transmission prolongs barbiturate-induced sleep. Haloperidol, a typical antipsychotic is reported to potentiate barbiturate-induced sleep, by decreasing the activity of the nigrostriatal and mesolimbic dopaminergic system involved in cortical activation and behavioral arousal and sensitizing the CNS to the depressant action of barbiturate.[18] In this study, pretreatment with MAE was found to prolong phenobarbitone-induced sleeping time in mice, suggesting that MAE, by decreasing dopaminergic transmission, increases the sensitivity of the CNS to the depressant action of phenobarbitone, which prolongs sleeping time.

Dopamine D_2_ receptors are predominantly present in rat vas deferens.[26] Dopamine produces dose dependent contractions of vas deferens. The result of the *in vitro* test indicates that MAE inhibits dopamine-induced contractions on rat vas deferens. Thus, it is concluded that MAE possesses antidopaminergic activity, mediated through dopamine D_2_ receptors.

In the acute toxicity test, MAE did not produce any detectable toxicity on oral and i.p. administration. No mortality was found, which is reflected by high LD_50_ of MAE.

## Conclusions

The present investigation concludes that the methanolic extract of *Morus alba* L.** leaves contains constituents that inhibit dopaminergic neurotransmission and possibly blocks dopamine D_2_ receptor. Thus, MAE possesses antidopaminergic activity. The results suggest that the leaves of *Morus alba* L. may have potential clinical application in the management of psychiatric disorders.
